# Grand Rapids

**Published:** 1898-05

**Authors:** 


### FIRST GRADUATES FROM THE GRAND RAPIDS VETERINARY COLLEGE.

The first commencement exercises of the Grand Rapids Veterinary College were held on the evening of March 25, 1898, at the college lecture hall, on Butterworth Avenue. There were six graduates and a large number of their friends were present and a number of the most prominent physicians of the city.

Dr. Dales presided, and Dr. Graves presented the diplomas. In his opening address Dr. Dales said:

Mr. President, Ladies, and Gentlemen: To-night is the inauguration of a new era in the medical history of Grand Rapids. The Grand Rapids Medical College, with its various departments, was organized less than one year ago. If you will glance at the history of medical schools you will find but very few of those most prosperous to-day which started on their career with a larger class of students from the beginning than this school established in our city. The importance of location, the clinical opportunities afforded, and the unusual interest manifested foreshadow for this institution unexpected success. This, the Veterinary Department of the Grand Rapids Medical College, is the first to complete its course of study for the year, and we meet here to-night to confer upon the successful members of this class that which they richly deserve—the degree of D.V.S., and the diploma awarded by this institution, signalizing to the world that this the first graduating class of the veterinary department have completed their studies as students of veterinary medicine, and are well worthy of the confidence which I hope may be bestowed upon them by a generous public.

It is our most earnest desire that this institution may ever be an honor to the gentlemen who pass from our midst as graduates, and that you can refer to your alma mater with a feeling of confidence and pride. I assure you this institution will have the interest of its students at heart, and be ever ready to lend a helping hand. The closing of this session brings to mind my own student days and the memorable night on which I received a roll of parchment, for it inspired the thought—all was conquered; but alas, it was not. This is but the beginning of a lifework, and our student days are never ended, and he who goes forth with the idea all knowledge has been attained is but dreaming, the truth of which he will very soon realize. Knowledge is power, and we should ever strive to attain it. Honesty of purpose and fair dealing with all mankind should be your motto.

The graduates are L. L. Conkey, M. T. Banasiewicz, George Harr, city; Thomas Bunberry, Niles; A. H. Swift, Freesoil; George H. Stevenson, Detroit.

Addresses were given by Major E. C. Watkins, Prof. C. N. Nye, Dr. J. B. Griswold, Rev. Dr. Gardner, Dr. D. M. Greene, Dr. L. E. Best, and L. L. Conkey.

Rewards were offered for the highest standing in examination, and these were awarded to Thomas A. Bunberry, gold medal; George Harr, apparatus for throwing horses; A. H. Swift, veterinary dictionary.

The exercises were very interesting and were considered a credit to the institution.

